# Visualizing chemical structure-subcellular localization relationships using fluorescent small molecules as probes of cellular transport

**DOI:** 10.1186/1758-2946-5-44

**Published:** 2013-10-05

**Authors:** Gus R Rosania, Kerby Shedden, Nan Zheng, Xinyuan Zhang

**Affiliations:** 1 Department of Pharmaceutical Sciences, University of Michigan College of Pharmacy, 428 Church Street, Ann Arbor, MI 48109, USA; 2 Department of Statistics, University of Michigan, Ann Arbor, MI 48109, USA

**Keywords:** Machine vision, Cheminformatics, Virtual reality, Data mining, Optical probes, Multivariate analysis, Human-computer interaction, Graphical user interface

## Abstract

**Background:**

To study the chemical determinants of small molecule transport inside cells, it is crucial to visualize relationships between the chemical structure of small molecules and their associated subcellular distribution patterns. For this purpose, we experimented with cells incubated with a synthetic combinatorial library of fluorescent, membrane-permeant small molecule chemical agents. With an automated high content screening instrument, the intracellular distribution patterns of these chemical agents were microscopically captured in image data sets, and analyzed off-line with machine vision and cheminformatics algorithms. Nevertheless, it remained challenging to interpret correlations linking the structure and properties of chemical agents to their subcellular localization patterns in large numbers of cells, captured across large number of images.

**Results:**

To address this challenge, we constructed a Multidimensional Online Virtual Image Display (MOVID) visualization platform using off-the-shelf hardware and software components. For analysis, the image data set acquired from cells incubated with a combinatorial library of fluorescent molecular probes was sorted based on quantitative relationships between the chemical structures, physicochemical properties or predicted subcellular distribution patterns. MOVID enabled visual inspection of the sorted, multidimensional image arrays: Using a multipanel desktop liquid crystal display (LCD) and an avatar as a graphical user interface, the resolution of the images was automatically adjusted to the avatar’s distance, allowing the viewer to rapidly navigate through high resolution image arrays, zooming in and out of the images to inspect and annotate individual cells exhibiting interesting staining patterns. In this manner, MOVID facilitated visualization and interpretation of quantitative structure-localization relationship studies. MOVID also facilitated direct, intuitive exploration of the relationship between the chemical structures of the probes and their microscopic, subcellular staining patterns.

**Conclusion:**

MOVID can provide a practical, graphical user interface and computer-assisted image data visualization platform to facilitate bioimage data mining and cheminformatics analysis of high content, phenotypic screening experiments.

## Background

Combinatorial libraries of prospective, organelle-targeted small molecule fluorescent probes have served as treasure troves of optical sensors of cell physiology [1-11]. Until recently, development of live cell imaging probes relied mostly on qualitative, manual microscopic observations of stained cells. With the invention of high content screening systems [12-18], microscopic imaging experiments can be automated, so that image data sets can be acquired from cells incubated with hundreds or thousands of candidate bioimaging probes, with the click of a button [5,14,16,17,19,20]. Nevertheless, the ability to identify trends in staining patterns across large numbers of probes and to link these trends to specific variations in the structure or physicochemical properties of the chemical agents has remained a major challenge. For bioimaging probe discovery and optimization efforts, data acquisition has been greatly facilitated by advances in combinatorial chemical synthesis and robotic screening technology as well as machine vision and cheminformatics analysis. Nevertheless, for bioimaging probe development, the ability to interpret machine vision and cheminformatics results based on visually recognizable, cellular staining patterns assessed by human viewers is essential.

Integrating advances in the cognate fields of cheminformatics and bioimage informatics [15,21-23], our research group has been developing computational tools to address the data mining challenges inherent to complex phenotypic screening experiments, such as bioimaging probe discovery and development efforts [6,24-27]. In 2003, we began cell-based screening experiments involving a combinatorial libraries of cell permeant, organelle-targeted fluorescent compounds [25]. For data mining, we began integrating machine vision [13,27-30] and cheminformatics approaches [13,24,27-31]. We demonstrated the ability to link the different chemical building blocks of small molecule bioimaging probes to the resulting staining patterns by applying multivariate regression approaches [24,28,29]. Independently, we developed predictive models linking the physicochemical properties of small molecule chemical agents to their subcellular distribution [30,32-34] to facilitate discovery and development of *in vivo*, site-directed bioimaging probes [35,36].

In spite of all these advances, the human observer still plays an important role in the discovery of new classes of bioimaging probes [27,29] because ultimately, staining patterns have to be recognizable, interpretable and mechanistically meaningful to the human eye. Furthermore, without visual validation, unsupervised image analysis can be affected by many potential imaging artifacts including focus drift, motion blur or the presence of insoluble precipitates or aggregates, which affects the interpretation of cell-associated staining patterns. On their own, machine vision algorithms may lead to misleading or erroneous interpretations because of inaccurate object segmentation, image registration, and other sources of error. Therefore, to facilitate visual inspection of large image data sets of probe screening experiments, we decided to construct MOVID: a practical, cheminformatics and machine vision-assisted, virtual reality-based image visualization platform, built from off-the-shelf hardware and software components. Here, we demonstrate how MOVID can be used to interpret the staining patterns of a combinatorial library of small molecule fluorescent probes of cellular transport, relating these patterns to the chemical structure, physicochemical properties and the predicted subcellular localization patterns of the probes.

## Experimental

### Acquisition of image data sets

Image data was obtained from HeLa cells incubated in the presence of 1,344 fluorescent styryl compounds synthesized by conjugating one of eight pyridinium or quinolinium groups (A-H) to one of 168 aldehyde groups (1-168) [27,28]. All 8 × 168 combinations were screened using a Kineticscan™ instrument (Cellomics, Inc., Pittsburgh, PA) to acquire images using the 20X objective of the instrument [27,28]. The cell permeant DNA stain Hoechst™ 33342 (“Hoechst”) was included in the media, as an orthogonal reference marker which labeled the cell nuclei to allow segmenting the individual cells in the images [27,28]. From each probe, twelve images were obtained. Six images were acquired after one hour incubation with the probe, and another six images where acquired after the probe was removed from the extracellular medium. The images were 512 × 512 pixels, with pixel intensities ranging from 0 to 4,095, corresponding to Hoechst, TRITC, FITC, and Cy5 channels (TRITC and FITC channels images were acquired with two camera exposure settings). For visualization, only the Hoechst™ and the 1 second exposure FITC, TRITC and Cy5 images were displayed. This image dataset was deposited in an open, public repository (http://deepblue.lib.umich.edu/; Query “HeLa cells incubated with styryl compound”).

### Calculation of quantitative image features associated with cellular staining patterns

Machine vision was used to analyze the cellular staining patterns of the styryl probes [27,28]. Briefly, the Hoechst channel images were used to identify the nuclear region of each cell. The resulting nuclear regions were then dilated by 10 pixels to create a cell mask. For analyzing styryl compound fluorescence, the images acquired with the FITC, TRITC and Cy5 channels were summed to create a total cellular intensity multichannel image corresponding to the signal of the styryl molecule fluorescence over the multiple acquisition channels [28]. Using the complement of the dilated cell region mask, the background pixel intensity of the multichannel image was measured. The median intensity value of the background pixels of each image was subtracted from the intensity value of every pixel in that image, and these adjusted intensity values were then truncated at zero. Images lacking styryl-specific fluorescence signal (e.g. with cellular fluorescence signal at or below the extracellular background fluorescence, or showing extensive saturation of pixel intensities) were excluded from further analysis. With images exhibiting above background cell-associated fluorescence, three key cell-associated image features capturing the optical signal intensity and staining patterns of the probes were measured [27,29]: 1) the ratio of cell associated pixel intensity/background pixel intensity; 2) the cytoplasmic-to-nucleus ratio of cell-associated pixel intensities; and, 3) the coefficient of variation of cell-associated pixel intensities.

### Cheminformatics analysis of structural, topological, and physicochemical property features

Physicochemical properties of styryl compounds and their building blocks were calculated with MOE (Molecular Operating Environment, Chemical Computing Group Inc., Montreal, Canada). Diversity with respect to quantitative properties was assessed by comparing histograms for each property between the styryl compounds and a reference dataset of hundreds of chemical agents with published subcellular localization features, compiled from a literature-based review surveying the organelle-targeting properties of small, drug-like molecules [30-32]. Diversity of chemical structures for the styryl molecules and for their chemical building blocks was assessed using Cactvs bit strings which represent chemical structures as 881- dimensional binary sequences. Each bit in a Cactvs sequence indicates the presence of a specific chemical substructure in the overall structure. The Tanimoto coefficient (Tc) was used to quantify the similarity between each pair of structures, by dividing the number of features in the Cactvs string that are common to both structures by the total number of features present in either structure. Mathematically, *Tc* = *C*/(*N*_1_ + *N*_2_ - *C*) where *N*_1_ and *N*_2_ correspond to the number of features present in the fingerprints of each molecule in the pair, and *C* is the number of features present in the fingerprints of both molecules.

### Predicting subcellular distribution patterns

The theoretical distribution of styryl molecules inside cells was calculated using a published mathematical model [30,33,37], based on the predicted passive diffusion properties of the compounds in the presence of a constant extracellular concentration of the compound. This mathematical model incorporates Fick’s law of diffusion, Henderson-Hasselbalch equation and the Nernst-Planck equation, to model the diffusion of small molecule chemical agents across the membranes bounding cellular / subcellular compartments, as determined by the concentration gradients, pH gradients, and electrical potentials across the bounding membranes. Computationally, the models allowed calculating the mass of compound that accumulates in various subcellular compartments, over time [30,33,34,38]. For simulating the transport behavior of the styryl molecules, all styryl molecules possess a single, fixed positive charge plus 0 or more additional ionizable groups. For this study, simulations were done only for molecules with 0 or 1 additional ionizable groups corresponding to 1256 out of 1344 molecules in the library (93.4%). Baseline input parameters of the model were based on an “average” mammalian cell using the established, generic physiological parameters of cells (such as membrane surface area, cellular and organelle volumes, pH values in each compartment, membrane potentials, and lipid fractions in each compartment) [33,39]. Input physicochemical properties of styryl molecules (such as logP and pKa) were calculated with Chemaxon (http://www.chemaxon.com). After the concentration at steady state was calculated for each compartment, the mass of styryl molecules in each compartment was calculated by multiplying the concentration in that compartment by the compartment’s volume. The total mass of compound in cell was then calculated as the sum of the masses in the different compartments: cytoplasmic (or plasma) membranes, lysosomes and mitochondria. The fractional mass of compound in cytoplasmic (or plasma) membranes, lysosomes and mitochondria was calculated based on the mass of compound in each compartment divided by the total mass of compound in these three compartments, at steady state.

### Developing machine vision- and cheminformatics-assisted MOVID platform

**Figure 1:**
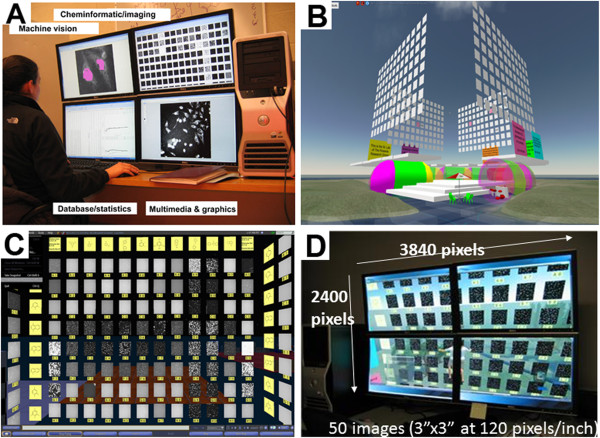
**MOVID integrates off-the-shelf hardware and software components to combine machine vision, cheminformatics and data visualization capabilities. A)** Multiscreen workstation set up allowed switching between different applications with the click of a mouse. **B)** Four-sided virtual image display constructed in Second Life. **C)** Frontal view of one of the four sides of the virtual image display. The background lighting has been dimmed and images are displayed in grey scale mode. Yellow panels contain image labels and chemical structure information. Blue bars at the bottom of the page are control buttons of the virtual reality browser. **D)** Picture of the multiscreen workstation, showing one of the image displays. The avatar is floating in the middle of the bottom two panels, between the screens, with the avatar’s head immediately below the intersection of the four screens.

Three dimensional virtual image displays were constructed in a multiuser, publicly accessible, online virtual reality software platform (Second Life, Linden Labs, Inc.) [40-43], Image arrays were manually constructed, or plotted with a PrimPlotter script (http://1cellpk.blogspot.com/). To facilitate visual inspection of image data sets at high resolution we used a desktop computer workstation (Dell, Inc) (Figure 1A). To simultaneously visualize images in multiple channels, four virtual image display data walls (front, back, left, right) were constructed perpendicularly to each other (Figure 1B). By placing an avatar in the middle of the four image display data walls, the entire image array could be readily visualized by moving the avatar away from the data wall, or a specific image could be visualized at higher resolution by moving the avatar towards the data wall (Figure 1C, D). Different acquisition channels or additional image arrays could be viewed simply by rotating the avatar, or by moving the avatar from one array to a neighboring array, or by changing the images displayed on the array. The display was designed so that it could simultaneously display the images of a 96 well screening plate, corresponding to 8 pyridinium or quinolinium building blocks conjugated to 10 aldehyde building blocks, as well as images from the outer two columns of control wells, labels for each image and chemical structure of the probes (Figure 1C). With this hardware and software set up, a 5 × 10 array of images could be simultaneously displayed at 120 pixels/inch resolution, with each image displayed at a size of 10 cm × 10 cm (Figure 1D).

## Results

### Analyzing the chemical diversity of the organelle-targeting styryl library

**Figure 2:**
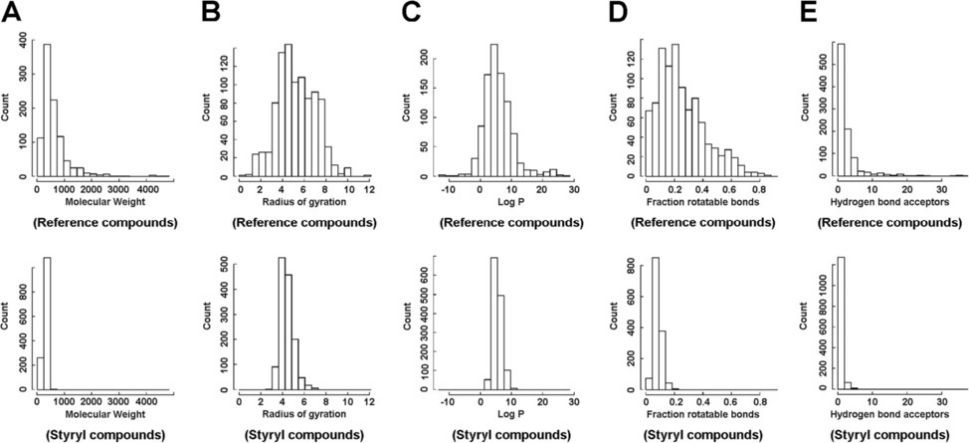
**Histogram plots comparing the calculated physicochemical properties of a reference dataset of compounds with known subcellular localization features (top row) in relation to the library of 1,344 styryl molecules analyzed in this study (bottom row). A)** Molecular weight; **B)** Radius of gyration; **C)** Logarithm of the octanol:water partition coefficients (logP); **D)** Fraction of rotatable bonds; **E)** Number of hydrogen bond acceptors.

All styryl compounds in the particular library analyzed in this study shared the same molecular scaffold and therefore possessed many features in common (Figure 2). Compared to a reference set of molecules with known subcellular localization features [31,32], cheminformatics analysis revealed that the styryl compounds were less diverse: they possessed a narrower range of molecular weights (Figure 2A); radius of gyration (Figure 2B); logP (Figure 2C); fraction of rotatable bonds (Figure 2D) and number of hydrogen bond acceptors (Figure 2E). More specifically, styryl compounds were < 500 Daltons in molecular weight; their radius of gyration ranged from 4 to 6 Armstrongs; their logP was generally between 2 and 6; their fraction of rotatable bonds tended to be < 0.2; and their number of hydrogen bond acceptors generally was <2. Among the 1344 styryl molecules, 872 compounds had one fixed positive charge and lacked additional ionizable groups. The rest of the compounds had a fixed positive charge plus one or two additional ionizable groups. By covering a smaller fraction of chemical space with many molecules of similar size, shape and chemical features, this focused combinatorial library of compounds allowed us to explore how small variations in topological features and chemical structures influenced cellular staining patterns [27].

### Characterizing the diversity of staining patterns in relation to the diversity of predicted localization patterns

**Figure 3:**
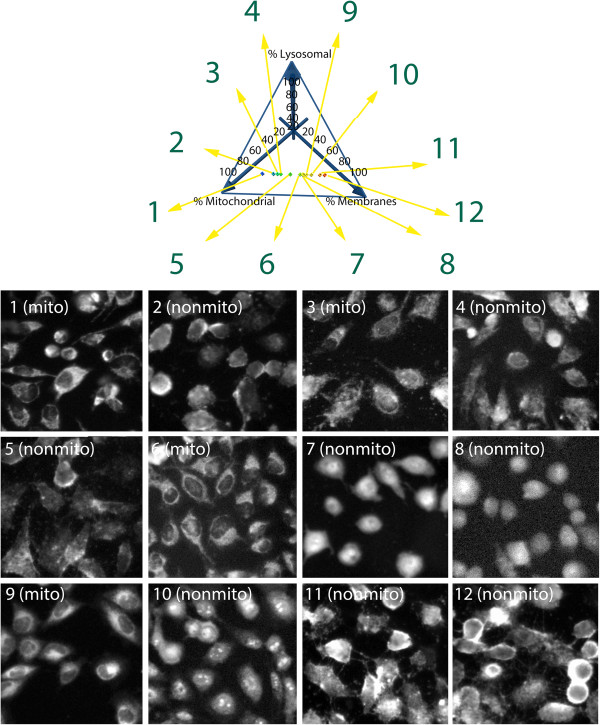
**Two dimensional projection of a three dimensional subcellular localization plot showing the predicted relative distribution of the styryl probes in lysosomes, mitochondria and cytosol.** In three dimensions, the origin of the graph would extend into the back of the page. Each axis projects towards the viewer (indicated by arrows). Each point in the plot represents an individual styryl molecule, according to its predicted, % mitochondrial, % lysosomal and % cytoplasmic (or plasma) membrane mass distribution. Each data point links to the chemical structure of each molecule and the images associated with each molecule including Hoechst, FITC, TRITC and Cy5 channels, the predicted localization and annotated staining patterns. Predicted localizations based on different input parameter values were visually compared to the staining patterns apparent in the images. Images and localization calls from 12 selected data points (numbered 1 – 12) are shown, in a range from predicted 100% mitochondrial localization to 100% cytoplasmic membrane localization, for a subset of compounds without significant lysosomal mass accumulation. Miner 3D (Miner 3D, Inc) was used to generate the figure. Additional images, chemical structures, and calculated properties for these twelve compounds are included in the (Additional file 1: Figure S1-S12).

Based on hypothetical relationships between lipophilicity, ionization and staining patterns, the distribution of the styryl compounds in different subcellular compartments was predicted using an established mathematical modeling approach [33,39]. For visualization purposes, the calculated localization patterns of each compound were plotted in three dimensions, with each axis corresponding to the percent mass localized in mitochondria, lysosomes, and other cytoplasmic (and plasma) membranes (Figure 3). In these 3D plots, each data point represented the predicted, percent mass accumulation of each molecule in these three compartments. Within each data point, the images associated with each molecule were displayed together with their 2D molecular structures, calculated physicochemical properties, and calculated subcellular concentrations (Additional file 1: Figures S1 to S12). Based on the simulation results, all styryl molecules were predicted to possess low lysosomal mass accumulation (less than 1% of the total mass of the molecule), with most compounds distributing between mitochondria and cytoplasmic (or plasma) membranes, reflecting their cationic, lipophilic character [33,39].

**Figure 4:**
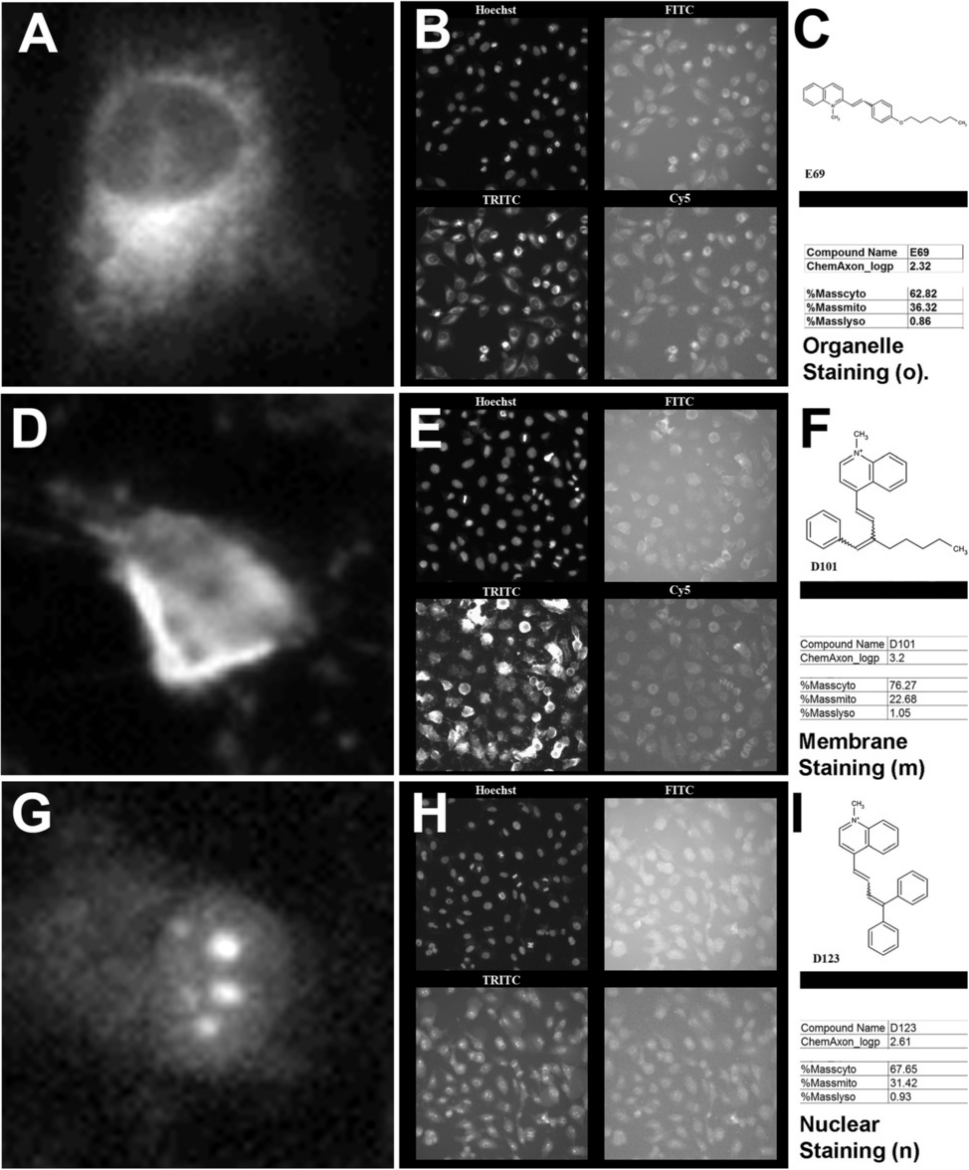
**Visual inspection revealed three distinct cell-associated staining patterns exhibited by the styryl probes. A)** Zoom in image of a single cell showing typical staining pattern of organelles. Note the nucleus in the middle is dark, and the surrounding cytoplasm is bright with local hot spots indicative of organelle staining. **B)** Hoechst (top left), FITC (top right), TRITC (bottom left) and Cy5 (bottom right) images corresponding to organelle staining probe. **C)** Chemical structure, logP and predicted cellular distribution data of the probe visualized in **A** and **B**. **D)** Zoom in image of a single cell showing typical staining pattern associated with cellular membranes. Note the probe distribution in the cytoplasm appears diffuse, with the brightest signal generally present at the cell periphery. **E)** Hoechst (top left), FITC (top right), TRITC (bottom left) and Cy5 (bottom right) images corresponding to membrane staining probe. **F)** Chemical structure, logP and predicted cellular distribution data of the probe visualized in D and E. **G)** Zoom in image of a single cell showing typical staining pattern of nuclear staining probe. Note the probe’s optical signal is brightest in the cell nucleus, with nucleolar labeling. **H)** Hoechst (top left), FITC (top right), TRITC (bottom left) and Cy5 (bottom right) images corresponding to nuclear staining probe. **I)** Chemical structure, logP and predicted cellular distribution data of the probe visualized in G and H.

However, after visually inspecting the image data set obtained with the styryl compounds, we established that many compounds with similar physicochemical properties and predicted subcellular distribution often exhibited different staining patterns (Figures 3, 4). For example, styryl molecule E69 (Figure 3, Compound 9) showed the typical organelle-associated staining patterns, characterized by a dark nucleus and bright cytoplasm with punctate foci (Figure 4A). The compound’s fluorescence signal was mostly associated with the TRITC channel (Figure 4B). The compound had a calculated logP of 2.32 and its predicted distribution was mostly in cytoplasmic (or plasma membranes (63%) and mitochondria (36%) (Figure 4C). In contrast, styryl molecule D101 (Figure 3, Compound 11) exhibited a pronounced membrane staining pattern (Figure 4D) which appeared brightest in the images acquired with the TRITC channel (Figure 4E). Nevertheless, its calculated logP and predicted distribution pattern was similar to that of styryl molecule E69 (Figure 4F). Another related styryl molecule, D123 (Figure 3, Compound 10), exhibited mostly nuclear staining (Figure 4G), with diffuse staining in the cytoplasm, and the fluorescence of signal of the compound being mostly detectable in the TRITC channel (Figure 4H). However, its logP and predicted subcellular localizations were similar to that of styryl molecules E69 and D101 (Figure 4I). Potentially, these differences in staining pattern may be reflecting the specific interactions between styryl molecules and cellular macromolecules, such as DNA or RNA, that are localized in specific subcellular compartments [6,24-27].

**Figure 5:**
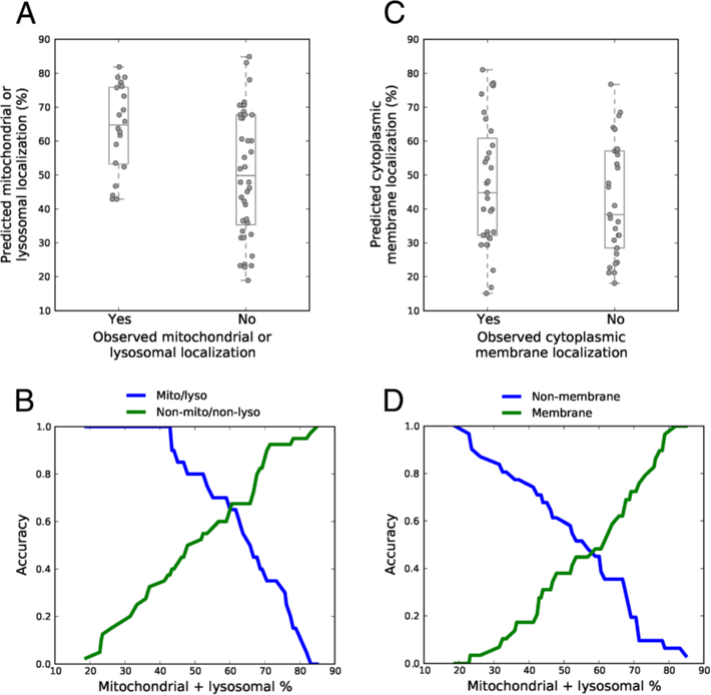
**The observed staining patterns in relation to the predicted, cellular staining patterns of styryl probes. A)** Plot shows the calculated, predicted percent accumulation of probes in mitochondria or lysosomes, relative to the observed staining patterns (YES means the compounds were visually scored as staining mitochondria or lysosomes; NO means the compounds were visually scored as staining cytoplasmic (or plasma) membranes, nuclei or nucleoli). **B)** The Accuracy of the predicted, subcellular distributions of probes to mitochondria or lysosomes relative to the observed mitochondrial or lysosomal (or nonmitochondrial and nonlysosomal) staining patterns, as shown in **A)**, plotted as a function of the threshold % predicted mass accumulation in mitochondria or lysosomes. Each threshold % mass accumulation is used to distinguish between those probes that are predicted to localize to mitochondria or lysosomes vs. those that are not. **C)** Plot shows the calculated, predicted percent accumulation in cytoplasmic membranes, relative to the observed staining patterns (YES means the compounds were visually scored as staining cytoplasmic or plasma membrane; NO means the compounds were visually scored as staining mitochondria, lysosomes nuclei or nucleoli). **D)** The Accuracy of the predicted, subcellular localization categories of the probes in cytoplasmic (or plasma) membranes relative to the observed cytoplasmic (or plasma) membrane staining pattern vs. other observed staining patterns as shown in **B)**, plotted as a function the threshold % predicted mass accumulation in mitochondria or lysosomes.

To quantitatively confirm these results, the predicted localization pattern of a subset of probes showing characteristic staining pattern was independently scored by an expert observer that was blind to the chemical structure and the predicted localization of the probes (Figure 5). For visual scoring, two pairs of binary localization categories were employed: (1) mitochondrial or lysosomal vs. non (mitochondrial or lysosomal); and, (2) cytoplasmic (or plasma) membrane vs. non (cytoplasmic (or plasma) membrane). From the observed staining patterns, we determined that when fractional mitochondrial or lysosomal mass accumulation was ≥40% there was an almost equal probability that the images were scored as being localized to (or not localized to) mitochondria or lysosomes, irrespective of the predicted percent accumulation (Figure 5A, B). Interestingly, when the predicted mitochondrial or lysosomal mass accumulation < 40%, all the compounds were scored as lacking a mitochondrial or lysosomal staining pattern (Figure 5A, B). Calculating the accuracy of the predicted localization to mitochondria or lysosomes revealed that the predictive accuracy for both mitochondrial or lysosomal and non (mitochondrial or lysosomal) was balanced (0.65) when the threshold, predicted mass distribution in mitochondria or lysosomes was 61% (Figure 5B). However, for the predicted cytoplasmic (or plasma) membrane localization, the number of molecules that were scored as cytoplasmic (or plasma) membrane staining were similar to those that were scored as staining either mitochondria, lysosomes, nuclei or nucleoli, irrespective of the predicted percent localization (Figure 5C, D). Again, the accuracy of the predicted localization to cytoplasmic (or plasma) membranes and to mitochondria, lysosomes, nuclei or nucleoli was balanced (0.47) when the threshold, predicted mass distribution in cytoplasmic membranes relative to other localizations was 59% (Figure 5D). Nevertheless, only those molecules that were predicted to show the least percent mass distribution in mitochondria or lysosomes showed direct correspondence to compounds that visually exhibited a non (mitochondrial or lysosomal) staining patterns. Accordingly, visual localization calls to mitochondrial or lysosomal or cytoplasmic (or plasma) membrane staining patterns generally did not show a strict correspondence to the calculated percent mass distribution of the styryl compounds in mitochondria or lysosomes vs. other cytoplasmic (or plasma) membranes.

### Visualizing relationship between chemical similarities and staining pattern similarities

**Figure 6:**
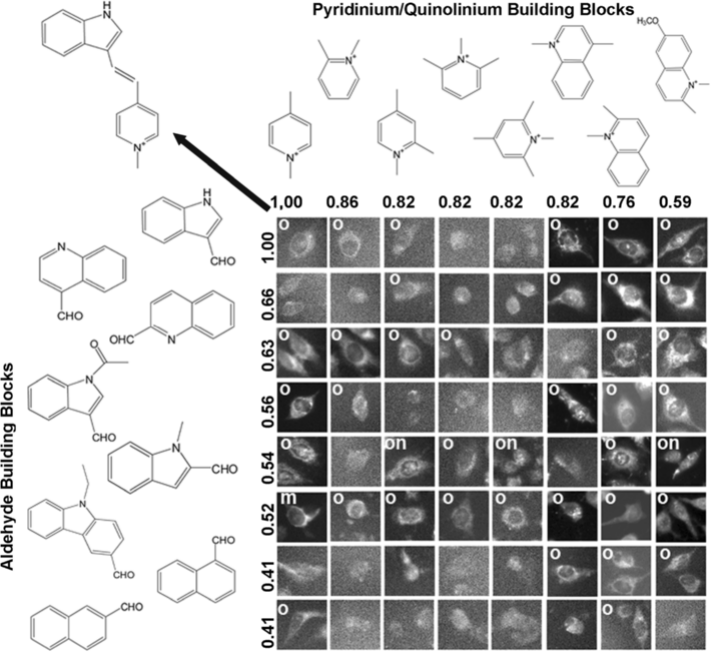
**Visualizing the relationship between the staining pattern of a reference probe (located at the upper left corner of the array; probe structure indicated by arrow) and the staining patterns of related compounds.** Different aldehyde building blocks are plotted in rows (left) and pyridinium or quinolinium building blocks are plotted in columns (top), in order of their calculated similarity to the building block of the reference compound on the top left. Numbers correspond to the Tanimoto coefficients between the different building blocks and the building blocks of the reference compound at the upper-left most corner of the array. To assemble figures, individual cells representing the staining patterns observed in each image were manually cropped from the images, and labeled based on their apparent organelle (o), membrane (m) or nuclear (n) staining patterns. Cells from images lacking significant signal or ambiguous in localization patterns were not labeled.

**Figure 7:**
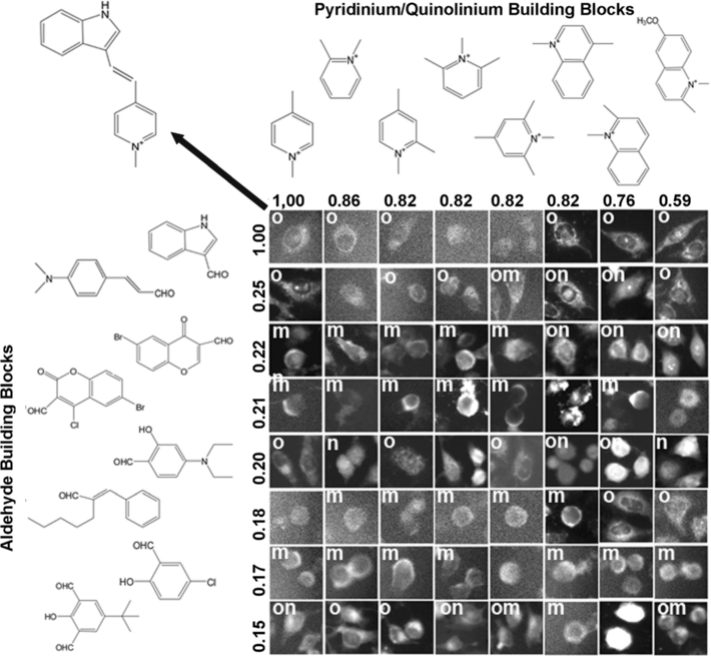
**Visualizing the relationship between the staining patterns of a reference probe (located at the upper left corner of the array; probe structure indicated by arrow) and the staining patterns of compounds with different chemical structures.** Different aldehyde building blocks were plotted in rows (left) and pyridinium or quinolinium building blocks were plotted in columns (top). Numbers correspond to the Tanimoto coefficients between the different building blocks and the building blocks of the reference compound at the upper-left most corner of the array. As in Figure 6, individual cells representing the staining patterns observed in each image were manually cut from the images, and labeled based on their apparent organelle (o), membrane (m) or nuclear (n) staining patterns. Cells from images lacking significant signal or ambiguous in localization patterns were not labeled.

A chemical fingerprinting approach was used to relate the structure of different pairs of probes based on shared similarities in the specific atoms and sub-fragments captured by the 2-dimensional connectivity of the chemical structure of the molecules. Intuitively, we expected that similar molecules would share similar staining patterns. Many styryl isomers in the library shared the same molecular formula and only varied in the *ortho-*, *meta-* and *para-* positions of attached functional groups. Therefore, we proceeded to visualize how staining patterns of styryl molecules were related to the staining patterns of specific reference compounds. For this purpose, two dimensional image arrays were constructed. With the reference probe in the upper-left corner of the array, aldehyde and pyridinium or quinolinium building blocks were sorted based on their Tanimoto coefficient with respect to the building blocks of the reference probe (Figures 6 and 7). Images were scored based on the most clearly discernible staining patterns: mitochondrial or lysosomal (o; Figure 4A); cytoplasmic (or plasma) membrane (m; Figure 4D); or, nuclear or nucleolar (n; Figure 4G).

To demonstrate this analysis, we focused our attention on a styryl probe that was previously found to accumulate in mitochondria [44-46] and is one of the best characterized probes in the styryl library (Figures 6 and 7, upper left). To demonstrate how chemical structure variation relates to variation in staining patterns, we identified two subsets of compounds in the styryl library – one consisting of compounds that have similar structures relative to reference probe (Figure 6), and one consisting of compounds that have dissimilar structures relative to reference probe (Figure 7). The subset of compounds that are structurally similar to the reference probe consisted of those compounds in the library having both the pyridinium or quinolinium building block and the aldehyde building block with a Tanimoto coefficient >0.4 relative to the reference probe. By visual inspection, the staining patterns of these probes appeared similar to the organelle-associated staining pattern of the reference probe, although there was substantial variation in the overall intensity (Figure 6). The subset of compounds deemed least similar to the reference probe consisted of compounds whose aldehyde building block had a Tanimoto coefficient <0.25 relative to the aldehyde building block of the reference probe. For these compounds, the staining patterns appeared to be different from the staining patterns of the reference probe (Figure 7). By visual inspection, the staining patterns of the probes revealed roughly similar numbers of membrane- and organelle- staining probes, and a smaller fraction of nuclear-staining probes. Based on the Tanimoto coefficients, the transition between visually similar vs. different staining patterns in relation to the Tanimoto coefficients appeared to be abrupt and nonlinear, with most compounds possessing an aldehyde group with a Tanimoto coefficient >0.4 exhibiting similar staining patterns as the reference probe (Figure 6). This observation may be an explanation for why previous, unsupervised machine vision and cheminformatics analyses did not reveal any significant correlation between Tanimoto coefficients and quantitative image features associated with the staining patterns [27].

### Visualizing relationship between staining patterns and regressed contributions of building blocks to staining patterns

**Figure 8:**
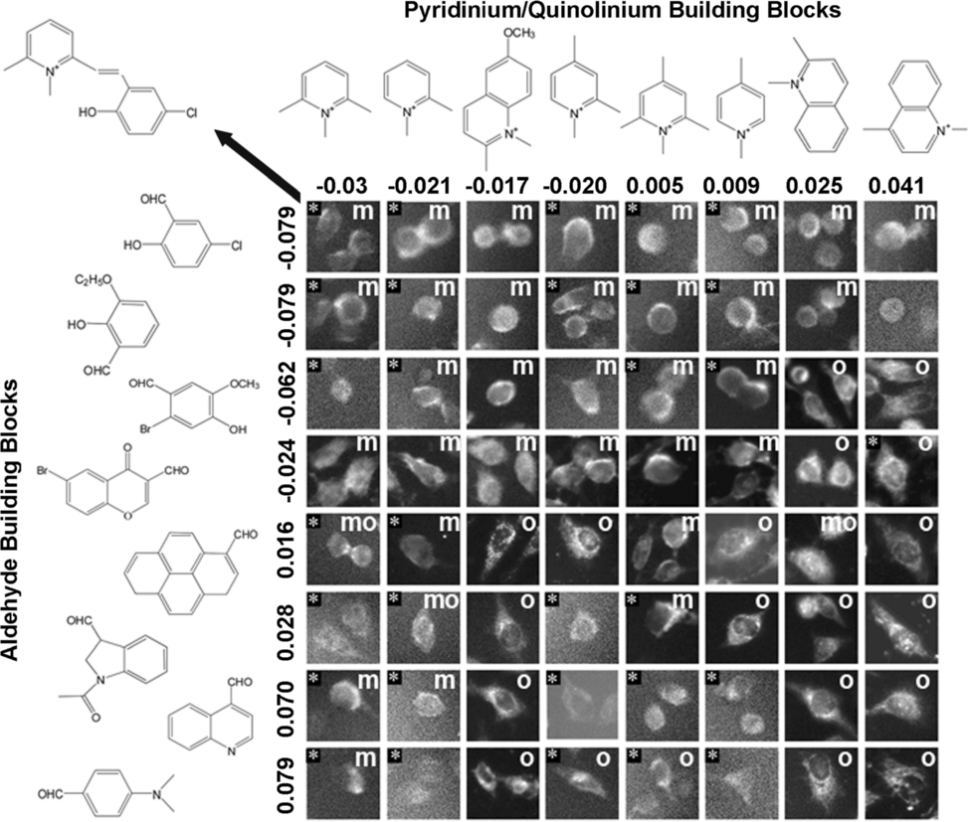
**Visualizing the relationship between cellular staining patterns and the calculated additive regression coefficients associated with each of the building blocks.** Different aldehyde building blocks were plotted in rows (left) and pyridinium or quinolinium building blocks were plotted in columns (top). The numbers indicate the magnitude and sign of the regression coefficient, based on the measured coefficient of variation of cell associated pixel intensities. To assemble figures, individual cells representing the staining patterns observed in each image were manually cut from the images, and labeled based on their apparent organelle (o), membrane (m) or nuclear (n) staining patterns. Cells from images lacking significant signal or ambiguous in localization patterns were not labeled. Images that were excluded from regression analysis based on saturation or lack of cell-associated signals were included for visualization and tagged with an asterisk (*). The arrow points to the structure diagram of the styryl compound corresponding to the image in the upper left hand corner of the array.

Previously, we used a quantitative multivariate regression approach to analyze relationships between chemical structures and image features [24,28]. Using a cross-validation approach, multivariate regressions results revealed that the aldehyde and pyridinium or quinolinium building blocks contributed to the cell-associated staining patterns in a predictive, additive manner [24,28]. To visually confirm these results, we proceeded to assemble image arrays based on sorted contributions of aldehyde and pyridinium or quinolinium building blocks (Figure 8). Based on diffuse vs. punctate staining patterns (captured by the coefficient of variation of cell associated pixel intensity) visual inspection of the aldehyde building block contributions to the staining patterns revealed a clear relationship between negative coefficients and diffuse, cytoplasmic (or plasma) membrane associated staining patterns (Figure 8 rows 1-4) and positive coefficients and punctate (mitochondrial or lysosomal) organelle-associated staining patterns (Figure 8, rows 5-8). Similarly, visual inspection of the pyridinium or quinolinium building block contributions to staining patterns confirmed the relationship between neutral or negative coefficients and diffuse, cytoplasmic (or plasma) membrane associated staining patterns (Figure 8, columns 1-6), and positive coefficients and positive (mitochondrial or lysosomal) organelle-associated staining patterns (Figure 8, columns 8 and 9).

## Discussion

In this study, we integrated the results of machine vision and cheminformatics analysis, with an image data visualization approach (MOVID), to facilitate visual interpretation of quantitative relationships between the chemical structure of a combinatorial library of small molecule fluorescent probes of cellular transport and their associated, cellular staining patterns captured in a large image data set. With MOVID, the ability to visualize multidimensional relationships was enhanced by allowing human viewers to literally navigate through sorted, high resolution image data sets, with the aid of an avatar. While at the level of the entire image, the ability to resolve local subcellular staining patterns is lost (Figure 4B, E, H), zooming into the individual images revealed details of the fluorescence signal in nucleus and cytoplasm of the individual cells (Figure 4A, D, G) that could be interpreted in the context of the chemical structure, physicochemical properties, and predicted localizations of the molecules. In this manner, MOVID facilitated interpretation of complex relationships between the phenotypic staining patterns of the cells and the chemical properties of the probes.

Using MOVID, we related the predicted, percent mass distribution of styryl molecules in different subcellular compartments to their observed staining patterns. When viewed across a large number of probes ranked based on their percent mass distribution in lysosomes, mitochondria and cytoplasmic (or plasma) membranes, a strict correspondence between predicted and visual localization patterns was not readily apparent (Figure 3). To further explore this relationship between the predicted localization patterns and observed staining patterns, the images obtained with the styryl compounds were visually classified by an expert observer (Figure 4). These observed staining patterns were used to calculate the accuracy of the predictions (Figure 5). Results of this analysis confirmed the original, qualitative visual impression (Figure 3), in that the predicted mass distribution of the probe in different compartments generally did not correspond with specific staining patterns. Only those compounds predicted to have a mitochondrial or lysosomal mass distribution of 40% or less showed a consistent association with the expected, nonmitochondrial and nonlysosomal staining pattern.

In addition, MOVID allowed us to confirm previous results obtained through machine vision and cheminformatics analyses [27,28]. For example, by visual inspection, similarities in the calculated physicochemical properties and subcellular distributions of the probes did not correspond to similarities in their subcellular localization patterns captured in the images (Figures 6, 7). Only compounds with *Tc* < 0.25 in the aldehyde building block (Figure 6) yielded clear visual differences in staining patterns as compared to the reference probe, explaining the absence of quantitative correlation between variations in *Tc* and variations in image features [27]. Furthermore, in the regression analysis, visual inspection of cellular staining patterns (Figure 8) also confirmed previous machine vision results [28] in terms of the ability of aldehyde and pyridinium or quinolinium building blocks to exert a predictive, additive contribution to diffuse vs. organelle-associated staining patterns, apparent across large numbers of images.

In light of previous machine vision and cheminformatics analyses [24,28], we also noted that the range in chemical diversity encoded by the variations in pyridinium or quinolinium building blocks was less than the range of chemical diversity encoded by variations in aldehyde building blocks (Figure 2). This corresponded to a more limited range in of regressed contributions to the staining patterns. Visual inspection of image arrays with MOVID were consistent with previous quantitative image analysis studies [28], indicating that chemical variations in aldehyde building blocks exerted a similar effect on the staining patterns as compared to chemical variations in the pyridinium or quinolinium building blocks. Previously, quantitative image analysis indicated that a charge push-pull mechanism affecting the electron distribution between the imminium group of the pyridinium or quinolinium and an amine group present in the opposite side of the molecule could exert a strong effect on quantitative image features [28]. Observed trends spanning large numbers of images were consistent with electron migration across the central methine bridge of the styryl molecule being associated with the diversity of staining patterns exhibited by styryl molecules. Interestingly, logP calculations generally do not take the effects of charge migration into account, which could partly explain the discrepancies between the predicted distribution of the probes and the observed staining patterns, with the exception of nonmitochondrial and nonlysosomal localizing compounds (Figures 3, 4 and 5).

In terms of the integration of off-the-shelf hardware and software components, the virtual, graphical user interface of MOVID offered distinct advantages over larger multipanel LCD arrays for high resolution image data display, also known as “command and control centers” or “data walls” (e.g. http://www.bioimage.ucsb.edu/iWall). MOVID was a practical, user-friendly and ergonomic alternative to display large amounts of visual information at high resolution, over a large field of view, to an individual user sitting at a desk. With the aid of an avatar, a viewer was able to rapidly scan through large numbers of images at very high resolution from a very close (virtual) distance, or at lower resolution from a farther (virtual) distance away (Figure 1). While sitting at a desktop computer workstation, a user can switch between MOVID to cheminformatics or modeling software with the click of the mouse (Figure 1A). In the past, many health care simulation, crowd-sourcing, open science, scientific conferencing and educational applications have been facilitated by the multiuser capabilities of online, open source virtual reality browsers [40-43]. Similarly, the open virtual reality graphics and multiuser interface capabilities of MOVID could also facilitate simultaneous, multiuser visualization capabilities at minimum cost.

## Conclusions

To conclude, the integration of cheminformatics and machine vision with a high resolution, multidimensional virtual reality image display and graphical user interface helped us overcome a major bottleneck we encountered while attempting to analyze the results of a phenotypic cell based assay. The associated data visualization and interpretation challenges are exemplified by the analysis of chemical determinants of the subcellular distribution properties of fluorescent small molecules probes of cellular transport, as elaborated in this study. In particular, despite their limited chemical diversity, our data was consistent with styryl molecules displaying a broad range of localization features, including probes that selectively labeled nuclei [26], nucleoli [6], plasma membrane [47] as well as other components [48,49]. Because bioimaging data is ultimately interpreted by human observers, visualization remains an essential component of bioimaging probe discovery and development, Nevertheless, beyond bioimaging probes, MOVIDs should be broadly applicable to many other phenotypic screening experiments [5,8,10,20,50,51] and subcellular drug targeting and delivery studies [2,18,33,39].

## Methods

### Visual scoring of subcellular localization patterns

To study how the predicted subcellular distribution patterns of the styryl molecules were related to their observed staining patterns, a total of 199 distinct images possessing a visible fluorescence signal were visually scored by an expert human observer, based on three distinct categories: (1) mitochondrial or lysosomal staining pattern; (2) cytoplasmic (or plasma) membranes staining pattern; (3) nuclear or nucleolar staining pattern. Only images of compounds that could be unambiguously classified in terms of one of these three distinct categories were considered. In some images, rounded (detached, mitotic or dying) were also present, and the localization of the probe in those cells was ignored. Images containing only rounded cells were not included in this analysis. This resulted in a subset of 123 images that could be used to compare how the predicted localizations related to the visual staining patterns.

### Relating the predicted, mitochondria- or lysosome-targeting styryl molecules to their visual staining patterns

To determine the accuracy of the mitochondrial or lysosomal localization predictions, we focused on probes that exhibited a perinuclear, punctate, “organelle” associated staining pattern characteristic of the mitochondria or lysosome-targeting compounds. First, we established whether the calculated, percent mass distribution in lysosomes or mitochondria of each probe that was visually observed as having a mitochondrial or lysosomal staining pattern was (i) equal to or greater than a threshold, calculated percent mass distribution in lysosomes or mitochondria (*true positives*); (ii) lower than the threshold, calculated percent mass distribution in lysosomes or mitochondria (*false positives*). In parallel, we established whether the calculated, percent mass distribution in mitochondria or lysosomes for each probe classified as nuclear, nucleolar, cytoplasmic (or plasma) membrane staining was (iii) equal to or greater than the threshold, calculated percent mass distribution in mitochondria or lysosomes (*false negatives*); or (iv) less than the threshold calculated percent mass distribution in mitochondria or lysosomes (*true negatives*). Relative to any given predicted, percent distribution threshold in the range of 0 to 100%, those compounds that were either *true positives* or *true negatives* were labeled either as *correctly classified* compounds. Conversely, those compounds whose predicted classifications were not *true positives* or *true negatives* were labeled as *incorrectly classified* compounds. Accordingly, the Accuracy of mitochondrial or lysosomal percent mass distribution predictions was calculated relative to the observed staining patterns, based on the following equation:

(1) $$\begin{array}{l} \mathrm{Accuracy}=\left(\#\text{correctly}\;\text{classified}\right)\\ \;/\left(\#\text{correctly}\;\text{classified}+\#\text{incorrectly}\;\text{classified}\right) \end{array}$$

For the mitochondrial or lysosomal localization predictions, the Accuracy of the predictions was plotted as a function of continuous thresholds of predicted percent mass distribution of the compounds in mitochondria or lysosomes, in the range of 0 to 100%. Independently, for the cytoplasmic (or plasma) membrane predictions, the Accuracy of the predictions was plotted as a function of continuous thresholds of, predicted percent mass distribution of compounds in mitochondria or lysosomes in the range of 0 to 100%.

### Relating the predicted, cytoplasmic (or plasma) membrane-targeting styryl molecules to their visually-scored staining patterns

Independently, we also calculated the accuracy of cytoplasmic (or plasma) membrane localization predictions in relation to the observed, visual staining patterns. For images of probes that were visually scored as possessing a cytoplasmic (or plasma) membrane localization pattern, we first established whether the calculated, percent mass distribution of the probe in cytoplasmic (or plasma) membranes was (i) equal to or greater than a threshold calculated percent mass distribution of probe in the cytoplasmic (or plasma membrane) compartment (*true positives*); or ii) lower than a threshold calculated percent mass distribution of probe in cytoplasmic (or plasma) membrane compartment (*false positives*). In parallel, we established whether the calculated, percent mass distribution in cytoplasmic (or plasma) membranes for each probe observed as nuclear, nucleolar, mitochondrial or lysosomal staining was (iii) equal to or greater than the threshold calculated percent mass distribution in cytoplasmic (or plasma) membrane (*false negatives*); or (iv) less than the threshold calculated percent mass distribution in cytoplasmic (or plasma) membranes (*true negatives*). In turn, the Accuracy of the cytoplasmic (or plasma) membrane distribution predictions was assessed in relation to their observed visual staining patterns based on the number of *correctly classified* and *incorrectly classified* compounds, in an analogous manner as was done for the mitochondrial or lysosomal distribution predictions (using Eq. 1), and plotted as a function of continuous thresholds of predicted, percent mass distribution in mitochondria or lysosomes in the range of 0 to 100%.

### Visualizing relationships between building blocks and staining patterns

To visualize relationship between similarities in the chemical structure of the building blocks and the associated images acquired from styryl molecules synthesized from those building blocks, image data sets were sorted in 2-dimensional arrays, based on the ranked, pair-wise Tanimoto coefficients between aldehyde and pyridinium or quinolinium building blocks, relative to the building blocks of a single, reference compound. The building blocks were sorted, in ranked order of decreasing pair-wise Tanimoto coefficients, relative to the building blocks of the reference probe. With MOVID, the image associated with the reference probe was displayed in the upper left corner of the image arrays. The individual images acquired from the styryl probes (made from pair-wise combinations of aldehyde and pyridinium or quinolinium building blocks) were displayed in the row and column position corresponding to the rank-ordered Tanimoto coefficient of the respective building blocks, relative to the reference probe.

### Visualizing the contribution of the different building blocks to the resulting staining patterns

The extent to which the two building blocks of the styryl molecules were associated with the various measured image features was calculated using a multivariate regression approach [29]. Briefly, in the styryl library, each molecule is comprised of an aldehyde building block that is chemically conjugated with a pyridinium or quinolinium building block. Thus, quantitative image features characterizing the cell-associated intensity and spatial signal of bioimaging probes can be related to each building block, assuming a simple additive contribution of each building block to any measurable image feature. For example, for any given image feature Y (Y = total cellular intensity, or N/C ratio, or nuclear CV), the linear model f(Y) = A(i) + P(j) can fit, where f is a suitable transformation such as the logarithm or identity function, i is the index of the aldehyde building blocks (A), and j is the index of the pyridinium or quinolinium building blocks (P). For visualization, aldehyde and pyridinium/quinolinium building blocks were sorted based on their contribution to the fitted numerical image features. Images were displayed with MOVID, with the sorted aldehyde building blocks corresponding to the rows in the grid, and the sorted pyridinium/quinolinium building blocks corresponding to the columns.

## Abbreviations

MOVID: Multidimensional online virtual image display; LCD: Liquid crystal display; DNA: Deoxyribonucleic acid; RNA: Ribonucleic acid; FITC: Fluorescein isothiocyanate; TRITC: Texas Red Isothiocyanate.

## Competing interests

The authors declare that they have no competing interests.

## Authors’ contributions

GRR: Designed and executed the experiments, analyzed the data and wrote the manuscript. KS, NZ and XZ: Analyzed the data and wrote the manuscript. All authors read and approved the final manuscript.

## Supplementary Material

Additional file 1: Figures S1 Compound 1 (from Figure 3). **Figure S2.** Compound 2 from Figure 3. **Figure S3.** Compound 3 from Figure 3. **Figure S4.** Compound 4 from Figure 3. **Figure S5.** Compound 5 from Figure 3. **Figure S6.** Compound 6 from Figure 3. **Figure S7.** Compound 7 from Figure 3. **Figure S8.** Compound 8 from Figure 3. **Figure S9.** Compound 9 from Figure 3. **Figure S10.** Compound 10 from Figure 3. **Figure S11.** Compond 11 from Figure 3. **Figure S12.** Compound 12 from Figure 3. Click here for file
