# Supplementary material for: Visualizing chemical structure-subcellular localization relationships using fluorescent small molecules as probes of cellular transport
Source: J Cheminform. 2013 Oct 5;5:44. doi: 10.1186/1758-2946-5-44 (PMC3852740; doi:10.1186/1758-2946-5-44)
Supplement: Additional file 1: Figures S1 — Compound 1 (from Figure 3). Figure S2. Compound 2 from Figure 3. Figure S3. Compound 3 from Figure 3. Figure S4. Compound 4 from Figure 3. Figure S5. Compound 5 from Figure 3. Figure S6. Compound 6 from Figure 3. Figure S7. Compound 7 from Figure 3. Figure S8. Compound 8 from Figure 3. Figure S9. Compound 9 from Figure 3. Figure S10. Compound 10 from Figure 3. Figure S11. Compond 11 from Figure 3. Figure S12. Compound 12 from Figure 3. [file 1758-2946-5-44-S1.pdf]

SUPPLEMENTARY MATERIALS

Visualizing Chemical Structure-Subcellular Localization Relationships Using Fluorescent Small Molecules as Probes of Cellular Transport

Gus R. Rosania[1*], Kerby Shedden[2], Nan Zheng[1#] and Xinyuan Zhang[1#]

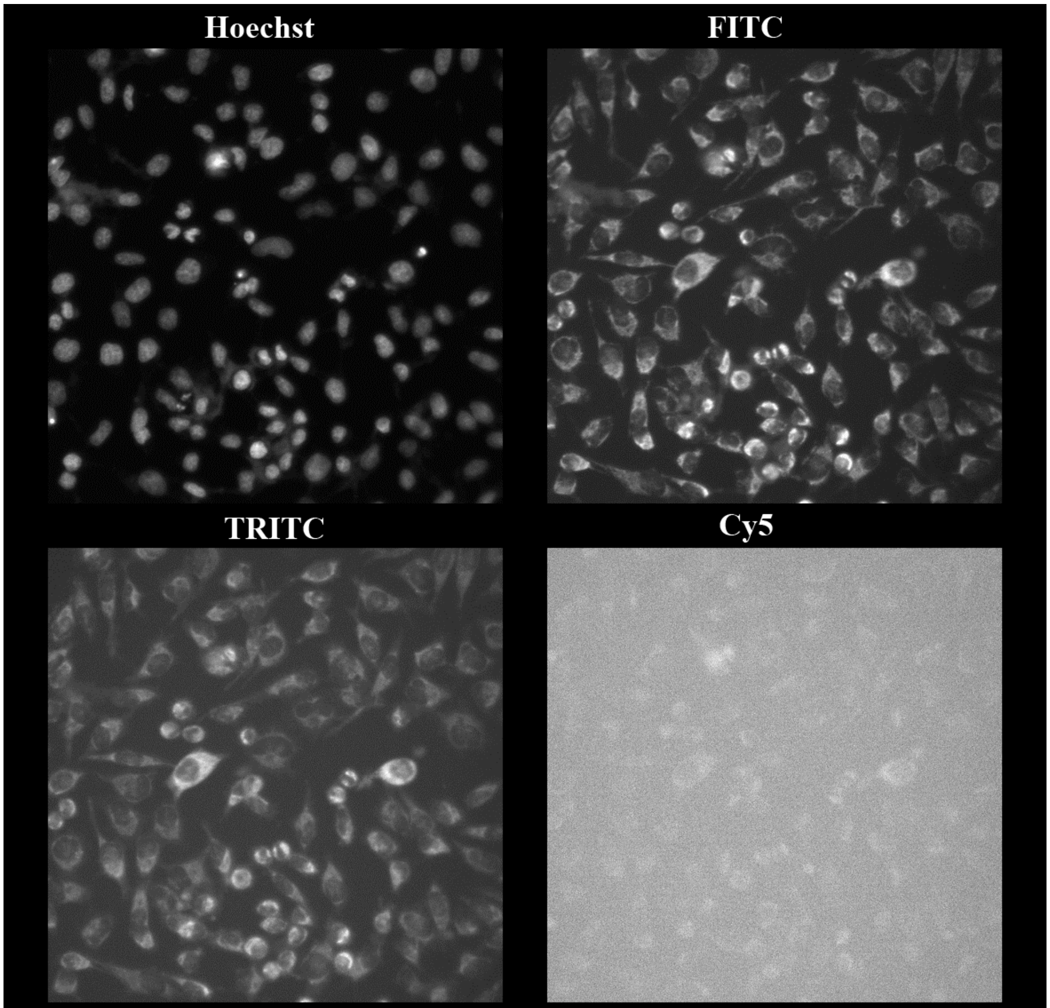

F153

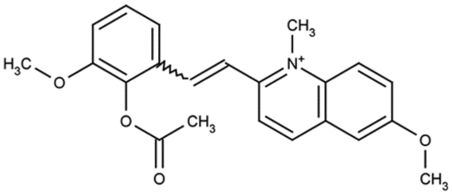

**Compound Name**	**F153**
**ChemAxon_logp**	**-0.44**
**logPd_lip**	**2.05**
**%Masscyto**	**18.28**
**%Massmito**	**81.46**
**%Masslyso**	**0.25**
**Localization**	**Mitolight**

Figures S1. Compound 1 (from Figure 3).

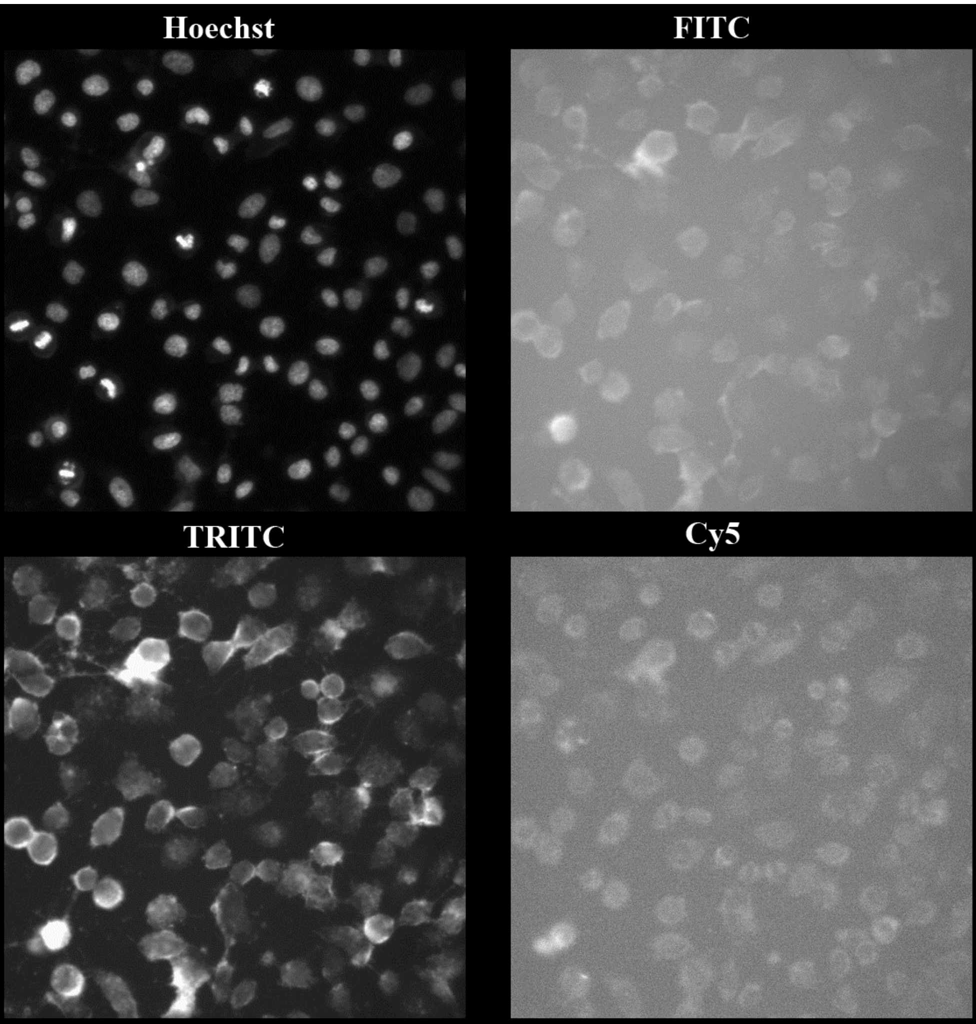

D102

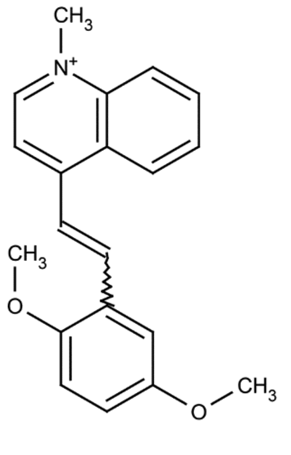

Compound Name	D102
ChemAxon_logp	0.42
logPd_lip	2.34
%Masscyto	29.40
%Massmito	70.20
%Masslyso	0.40
Localization	Plasma Membrane

Figure S2. Compound 2 from figure 3.

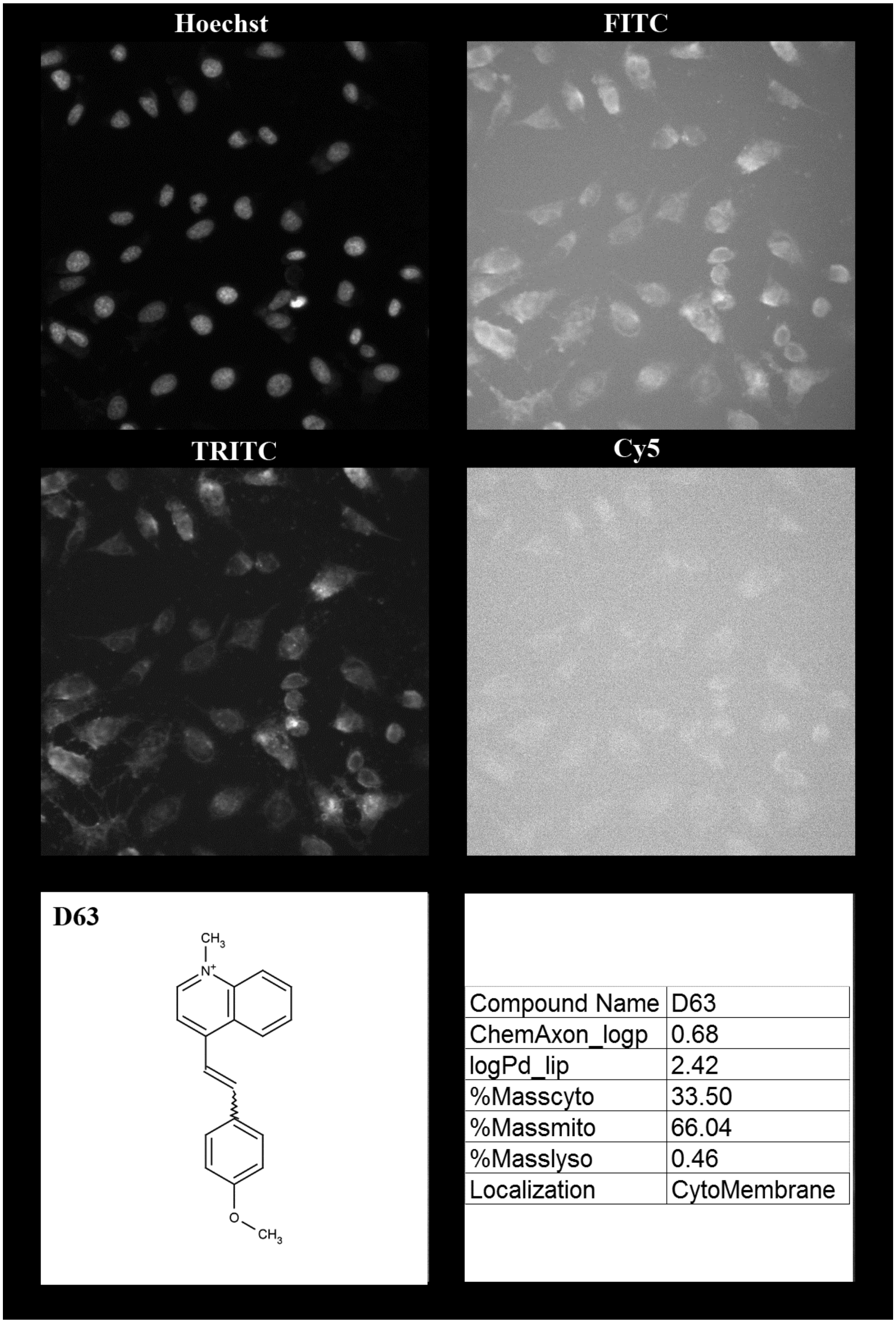

Compound Name	D63
ChemAxon_logp	0.68
logPd_lip	2.42
%Masscyto	33.50
%Massmito	66.04
%Masslyso	0.46
Localization	CytoMembrane

Figure S3. Compound 3 from Figure 3.

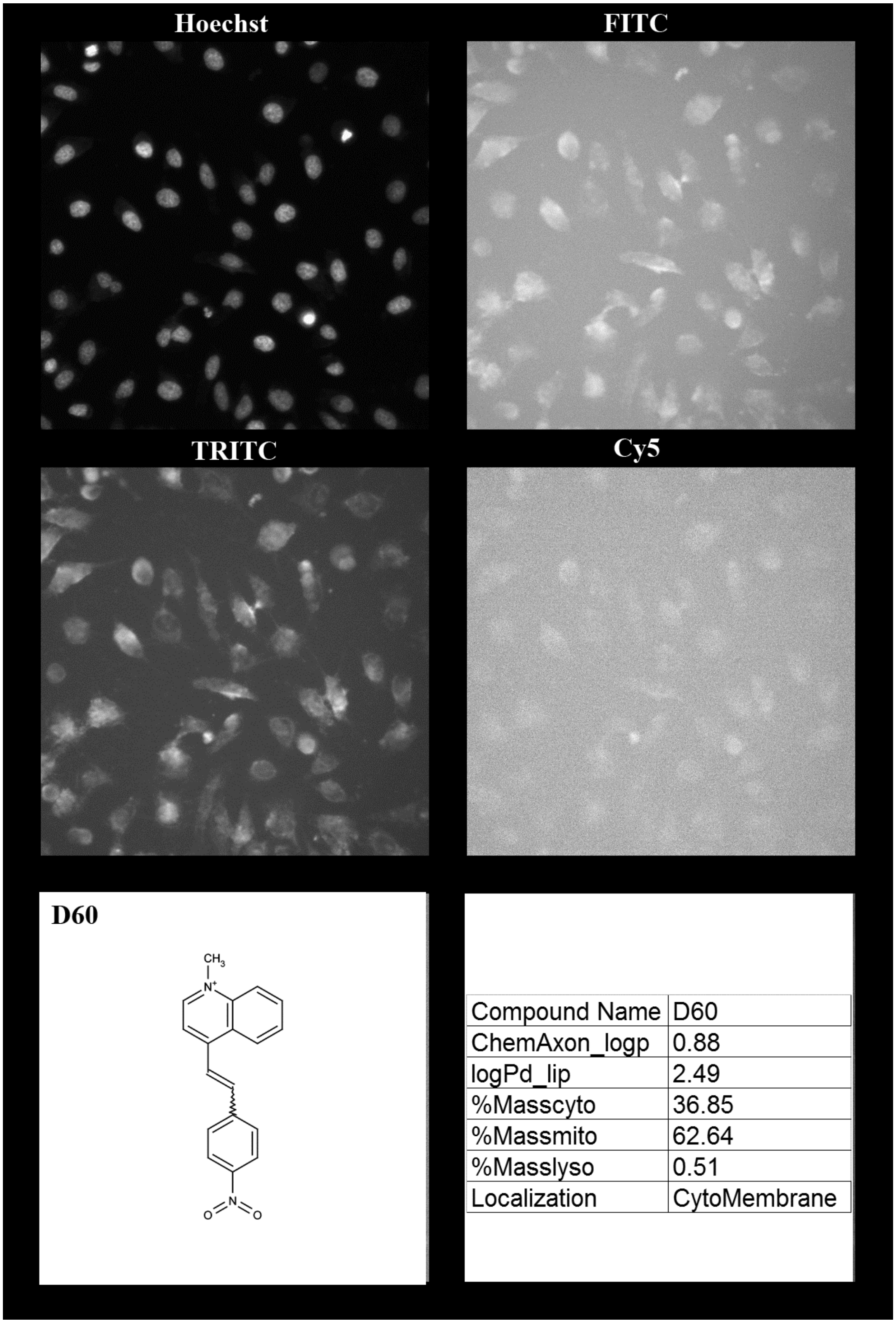

Compound Name	D60
ChemAxon_logp	0.88
logPd_lip	2.49
%Masscyto	36.85
%Massmito	62.64
%Masslyso	0.51
Localization	CytoMembrane

Figure S4: Compound 4 from figure 3.

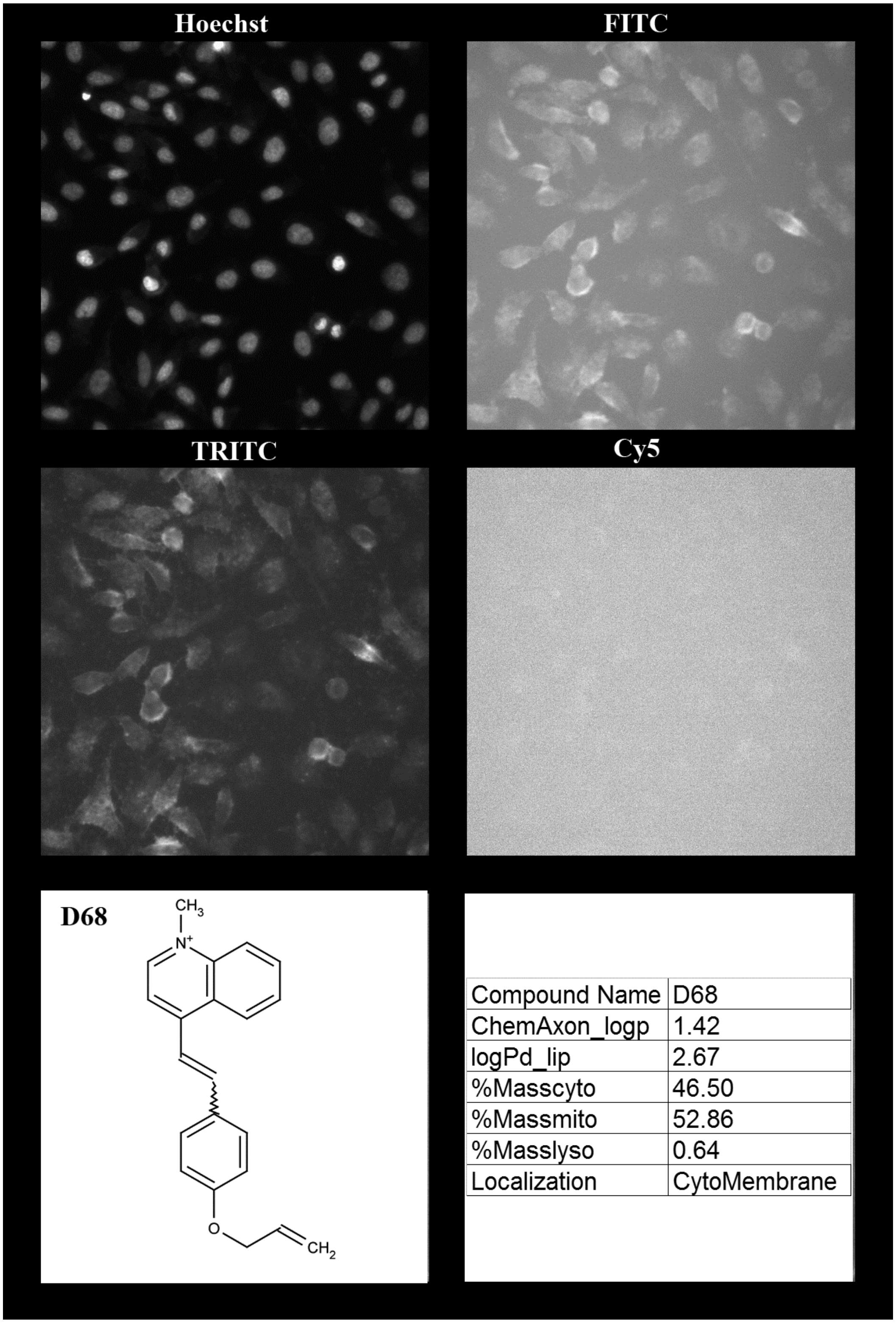

Compound Name	D68
ChemAxon_logp	1.42
logPd_lip	2.67
%Masscyto	46.50
%Massmito	52.86
%Masslyso	0.64
Localization	CytoMembrane

Figure S5. Compound 5 from figure 3.

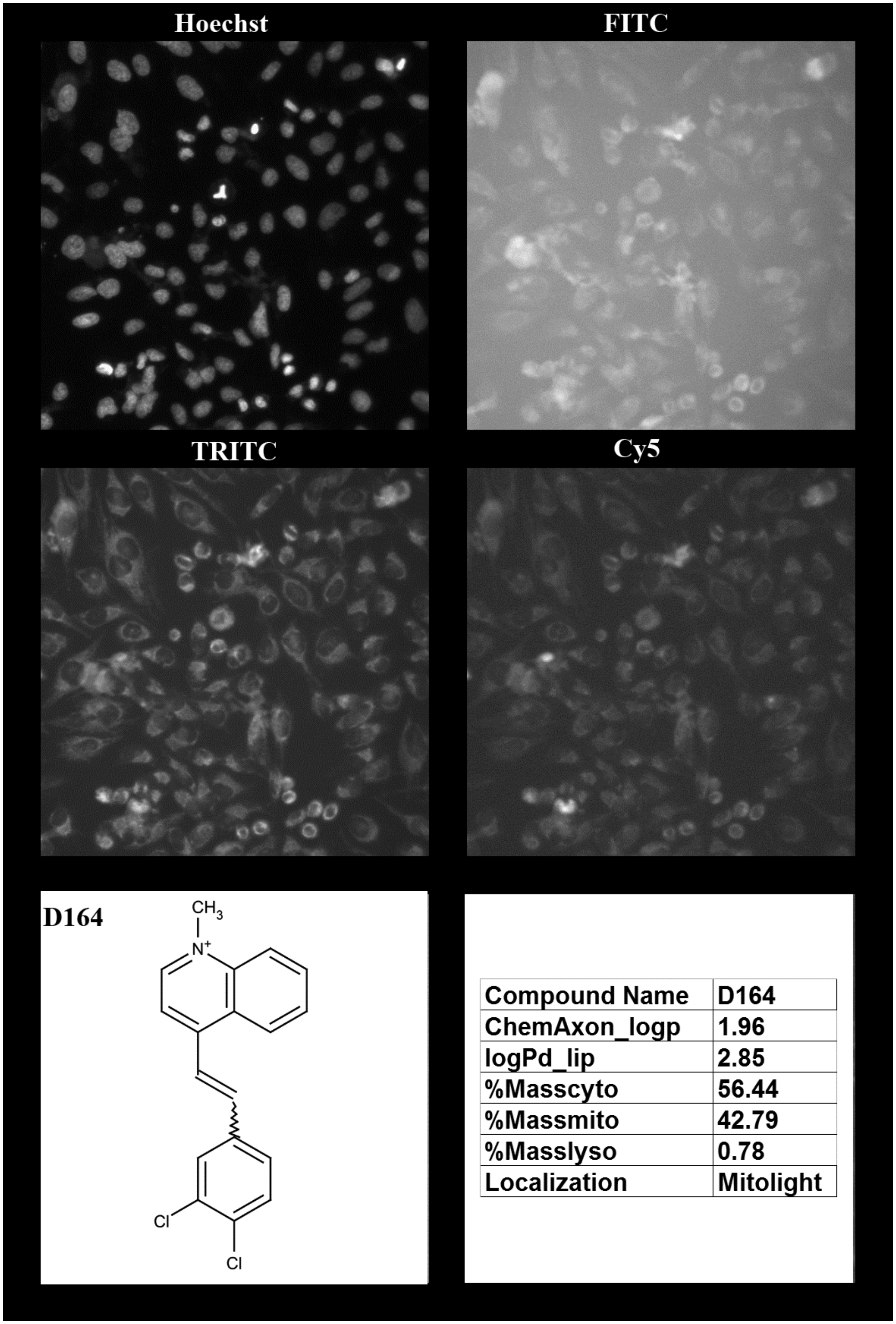

Compound Name	D164
ChemAxon_logp	1.96
logPd_lip	2.85
%Masscyto	56.44
%Massmito	42.79
%Masslyso	0.78
Localization	Mitolight

Figure S6. Compound 6 from figure 3.

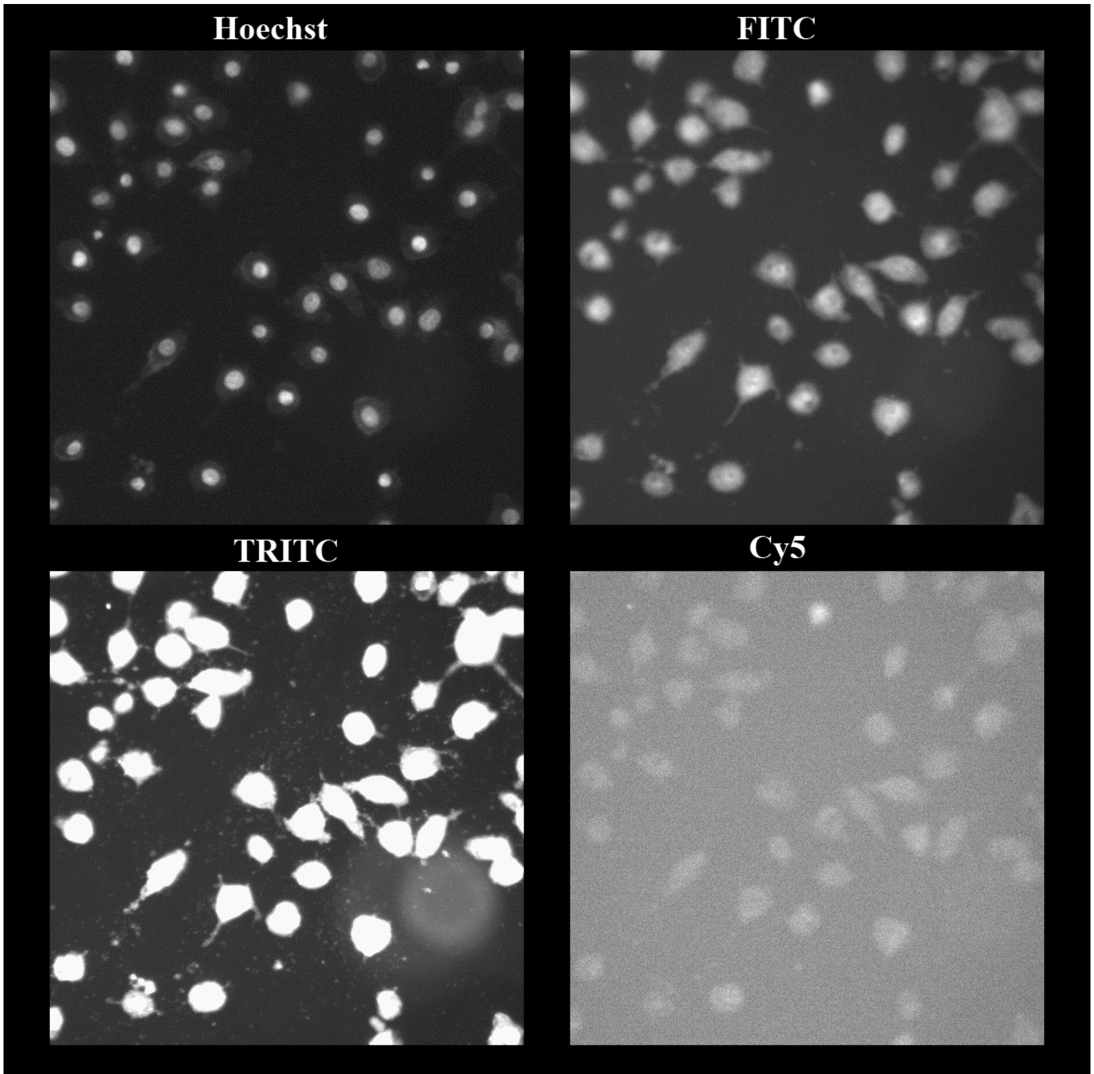

F107

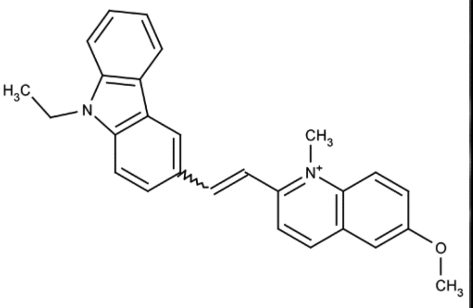

Compound Name	F107
ChemAxon_logp	2.1
logPd_lip	2.89
%Masscyto	58.96
%Massmito	40.23
%Masslyso	0.81
Localization	RNA

Figure S7. Compound 7 from figure 3.

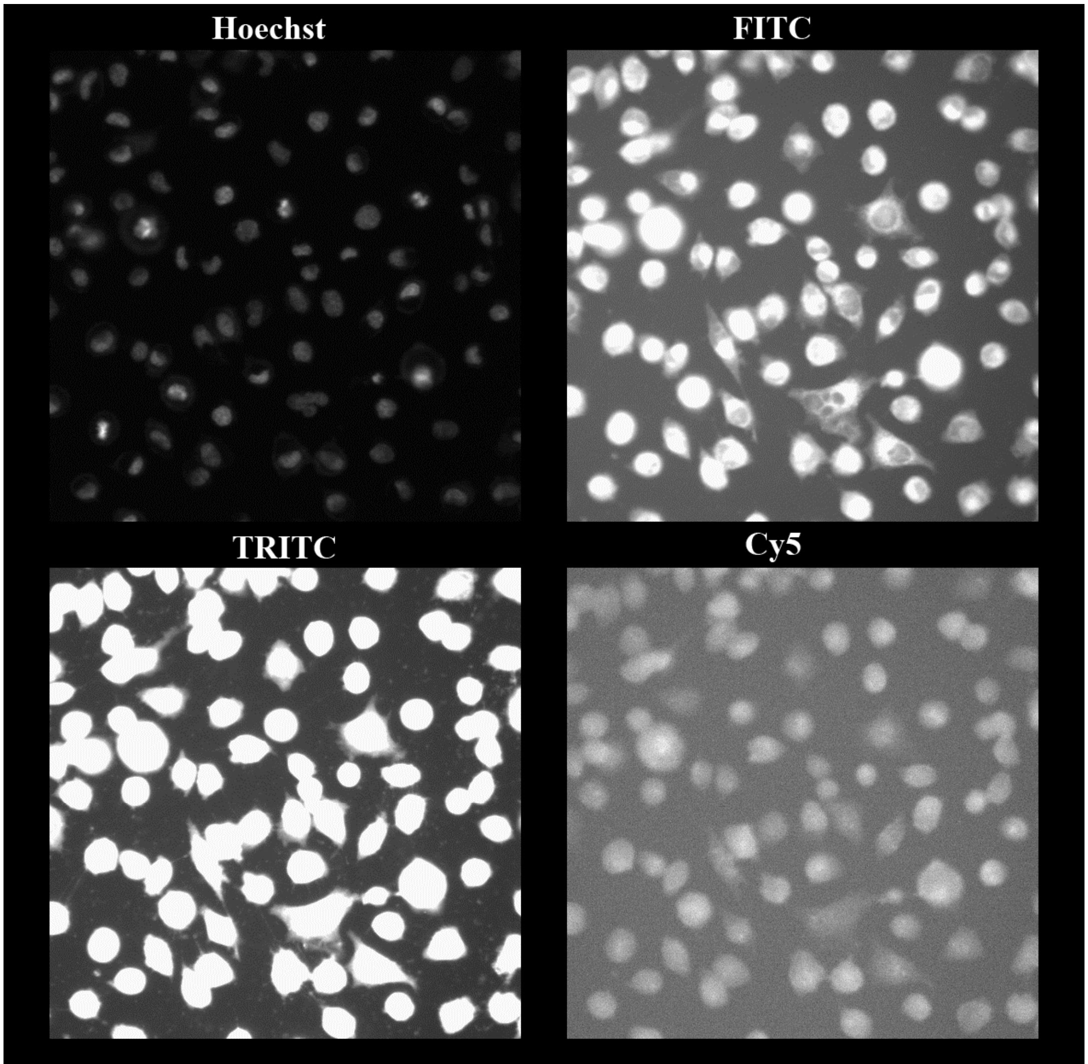

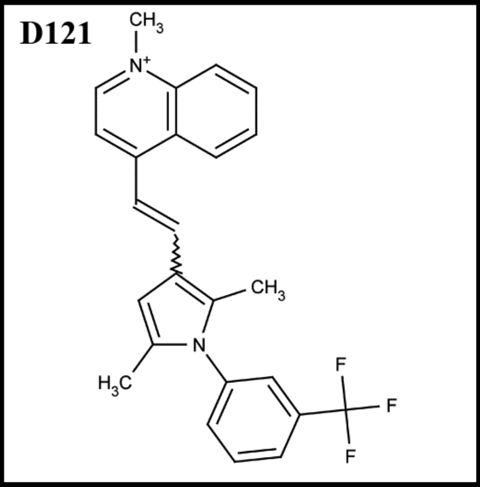

Compound Name	D121
ChemAxon_logp	2.16
logPd_lip	2.91
%Masscyto	60.03
%Massmito	39.15
%Masslyso	0.83
Localization	RNA

Figure S8. Compound 8 from figure 3.

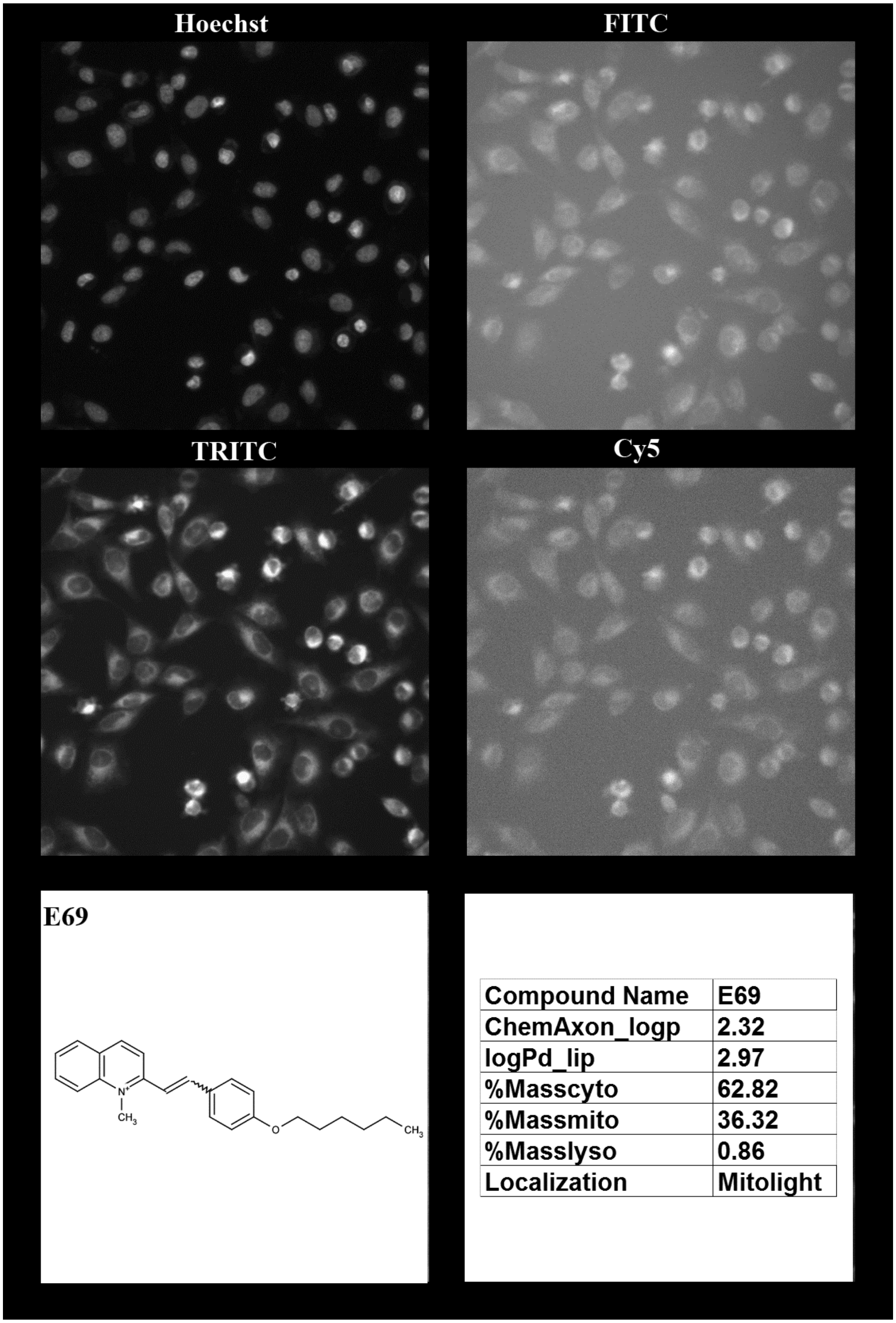

Compound Name	E69
ChemAxon_logp	2.32
logPd_lip	2.97
%Masscyto	62.82
%Massmito	36.32
%Masslyso	0.86
Localization	Mitolight

Figure S9. Compound 9 from figure 3.

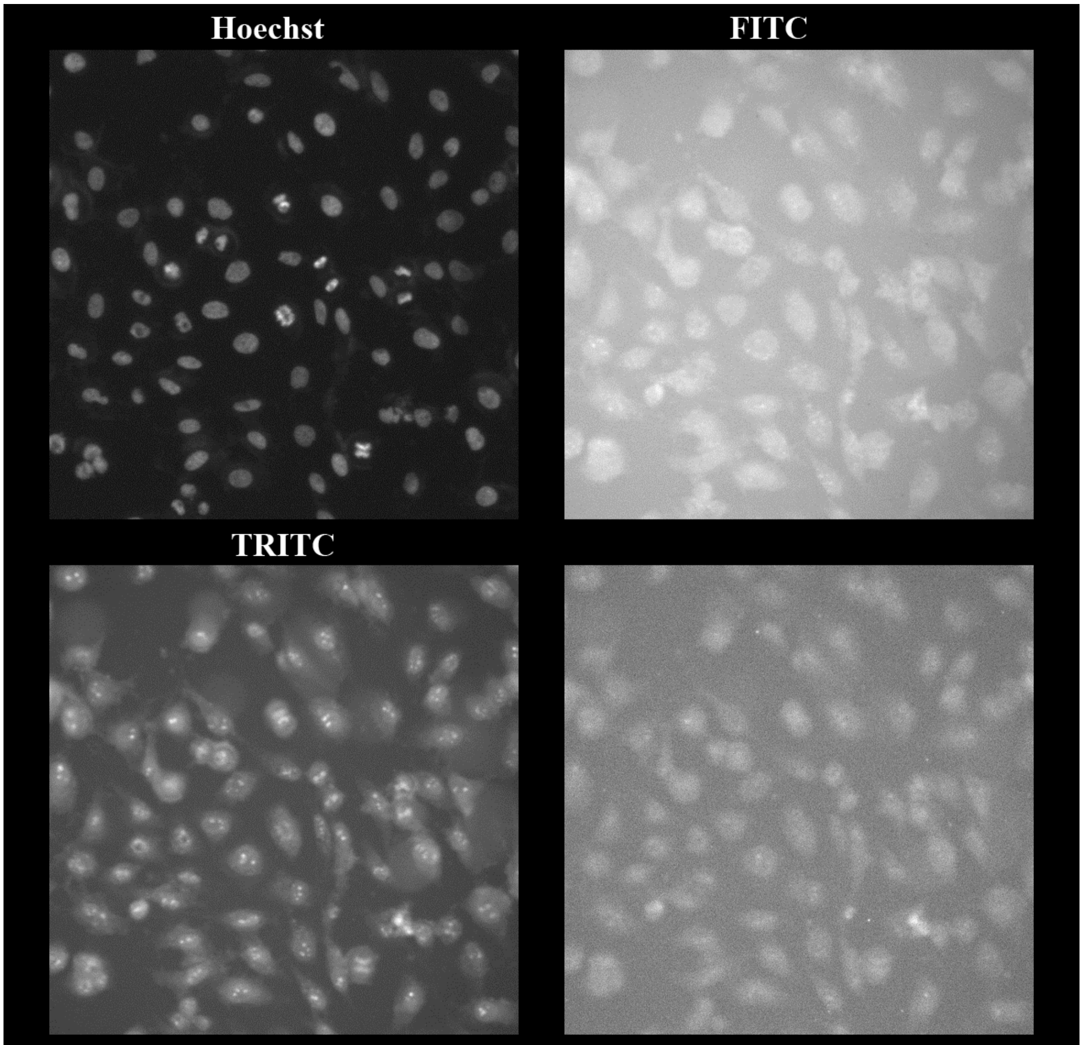

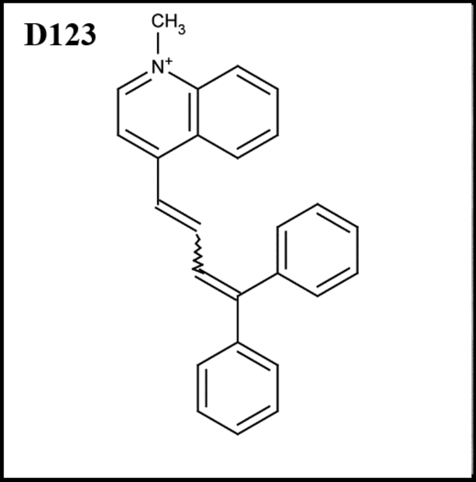

Compound Name	D123
ChemAxon_logp	2.61
logPd_lip	3.06
%Masscyto	67.65
%Massmito	31.42
%Masslyso	0.93
Localization	RNA

Figure S10. Compound 10 from figure 3.

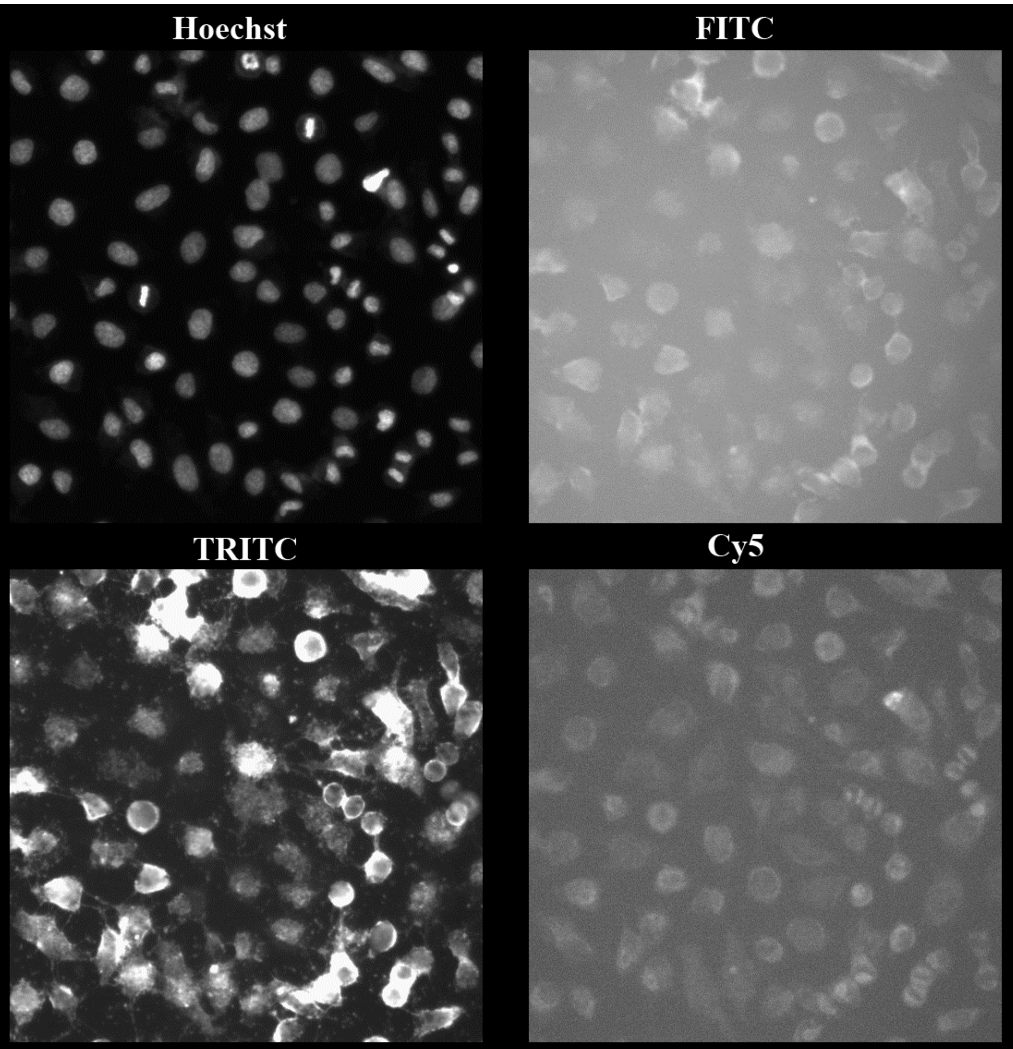

D101

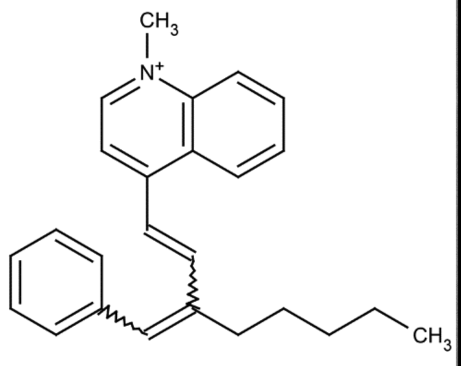

Compound Name	D101
ChemAxon_logp	3.2
logPd_lip	3.26
%Masscyto	76.27
%Massmito	22.68
%Masslyso	1.05
Localization	Plasma Membrane

Figure S11. Compond 11 from figure 3.

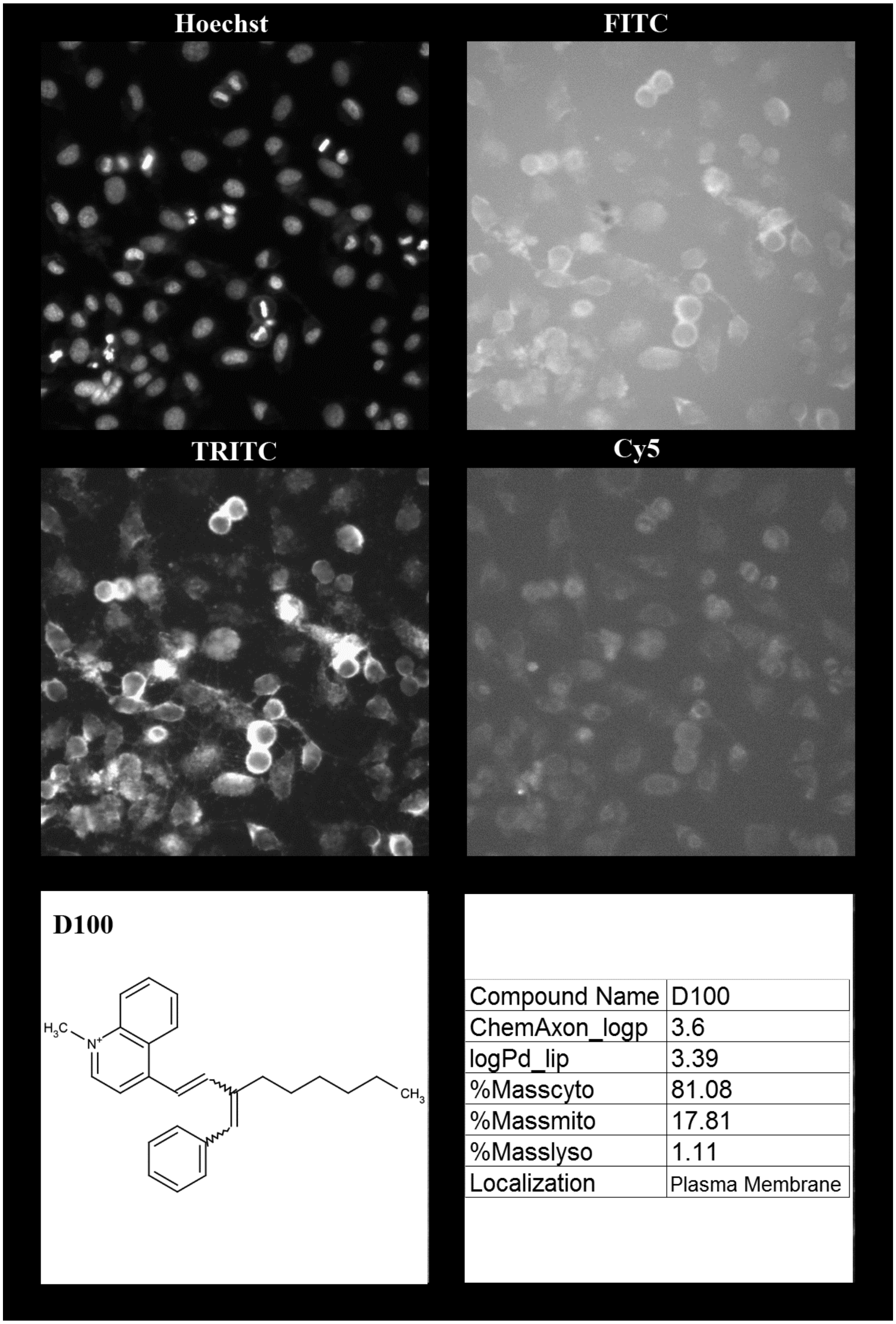

Figure S12. Compound 12 from figure 3.
